# Evaluation of saliva and nasopharyngeal swab sampling for genomic detection of SARS-CoV-2 in children accessing a pediatric emergency department during the second pandemic wave

**DOI:** 10.3389/fmicb.2023.1163438

**Published:** 2023-04-17

**Authors:** Erica Diani, Davide Silvagni, Virginia Lotti, Anna Lagni, Laura Baggio, Nicoletta Medaina, Paolo Biban, Davide Gibellini

**Affiliations:** ^1^Microbiology Section, Department of Diagnostic and Public Health, University of Verona, Verona, Italy; ^2^Pediatric Emergency Room, Department of Neonatal and Pediatric Critical Care, University of Verona, Verona, Italy; ^3^Microbiology Unit, AOUI Verona, Verona, Italy

**Keywords:** SARS-CoV-2, children, infection, pediatric, COVID-19, sample collection, nasopharyngeal swab, salivary swab

## Abstract

SARS-CoV-2 infection is mainly detected by multiplex real-time RT-PCR from upper respiratory specimens, which is considered the gold-standard technique for SARS-CoV-2 infection diagnosis. A nasopharyngeal (NP) swab represents the clinical sample of choice, but NP swabbing can be uncomfortable to the patients, especially for pediatric-age participants, requires trained healthcare personnel, and may generate an aerosol, increasing the intrinsic exposure risk of healthcare workers. The objective of this study was to compare paired NP and saliva samples (SS) collected from pediatric patients to evaluate whether the saliva collection procedure may be considered a valuable alternative to the classical NP swab (NPS) sampling in children. In this study, we describe a SARS-CoV-2 multiplex real-time RT-PCR protocol for SS, comparing the results with the paired NPS specimens from 256 pediatric patients (mean age 4.24 ± 4.40 years) admitted to the hospital emergency room of Azienda Ospedaliera Universitaria Integrata (AOUI), Verona, and randomly enrolled between September 2020 and December 2020. The saliva sampling demonstrated consistent results when compared to NPS use. The SARS-CoV-2 genome was detected in 16 out of 256 (6.25%) NP samples, among which 13 (5.07%) were positive even when paired SS were analyzed. Moreover, SARS-CoV-2-negative NPS and SS were consistent, and the overall concordances between NPS and SS were detected in 253 out of 256 samples (98.83%). Our results suggest that saliva samples may be considered a valuable alternative to NPS for SARS-CoV-2 direct diagnosis with multiplex real-time RT-PCR in pediatric patients.

## 1. Introduction

SARS-CoV-2, the causative agent of the COVID-19 disease, belongs to the *Coronaviridae* family and represents the third coronavirus that has emerged as a new human pathogen with severe clinical impact in the last 20 years, after SARS-CoV-1 and MERS-CoV infections (Braz-Silva et al., 2020; Santos et al., 2020; Sapkota et al., 2020; To et al., 2020). This virus has accounted for 755,041,562 infected patients and 6,830,867 deaths as of 8 February 2023 (https://covid19.who.int).

The dramatic impact of the SARS-CoV-2 infection has led to the use of specific measures to tackle virus transmission, including social distancing and lockdown strategies, which have significantly influenced the living conditions of people in large areas of the world (Al Suwaidi et al., 2021; Borghi et al., 2021). Moreover, continuous monitoring of the SARS-CoV-2 infection with molecular analysis has been determined an effective tool to control virus spread. Multiplex RT-PCR assay is considered the gold-standard technique for SARS-CoV-2 infection diagnosis (Liu et al., 2020; Tan et al., 2021) and is preferentially applied on nasopharyngeal swabs (NPS) (Borghi et al., 2021). This sampling approach is a valuable procedure, but it presents some concerns, such as coughing, discomfort, pain, and, in some cases, bleeding in patients (Melo Costa et al., 2021), especially in individuals with coagulation alterations. In particular, this sampling procedure shows lower compliance, which may discourage its use by some patients, especially children. Some reports have indicated saliva as an alternative specimen (Niedrig et al., 2018; Khurshid et al., 2020; Boutros et al., 2021; Poukka et al., 2021; Wang et al., 2021), suggesting different saliva sampling procedures to improve viral detection and encouraging people to self-test, thereby improving monitoring.

The advantages of a saliva sample (SS) compared to NPS for SARS-CoV-2 diagnosis are clearly indicated by the non-invasive collection method, reduced need for healthcare professional handling, and the possibility of self-collection and sampling, even outside the hospital. Several studies indicated that saliva sampling might be considered an alternative tool for the detection of SARS-CoV-2 sequences (Fan et al., 2021; Yee et al., 2021; Wyllie and Premsrirut, 2022), and some reports showed similar performances in SARS-CoV-2-specific multiplex RT-PCR when NPS and SS were compared (Ana Laura et al., 2021; Banerjee et al., 2021; Yee et al., 2021). SS may be very useful in pediatric patients since NPS has proven to be difficult to perform in children, especially those in the infant age. In this study, we analyzed a comparison between paired samples of NPS and SS collected from pediatric patients to evaluate the performances of these collection procedures in this diagnostic context.

## 2. Materials and methods

### 2.1. Study design and sample collection

A total of 256 pediatric patients were randomly enrolled between September 2020 and December 2020. These patients were admitted to the Pediatric Emergency Department of Azienda Ospedaliera Universitaria Integrata (AOUI), Verona. This study was approved by the local ethics committee (protocol 3442 CESC).

Paired nasopharyngeal and saliva samples were collected from each patient. NPS was performed using Copan E-Swabs (COPAN Brescia, Italy), following the current procedure. The saliva sample collection was performed with a cotton swab, adopting a procedure where the operator rolls the swab into the mouth and under the tongue. Samples were either sent to the virology laboratory within 1 h of collection or stored at 4°C. Samples were then immediately processed or stored at −20°C until analysis.

### 2.2. Multiplex real-time RT-PCR for SARS-CoV-2

The analysis of NPS and SS for the SARS-CoV-2 genome was performed using a commercial multiplex real-time RT-PCR platform. In brief, nucleic acids were extracted from samples using the Nimbus system (Nimbus, Seegene, Seoul, Korea). Amplification was performed using a COVID-19 kit (Seegene), following the protocol indicated by the manufacturer.

Purified RNA was amplified to detect different targets placed in E, RdRp/S, and *N* viral genes. The result was considered positive when at least one of these gene targets showed a cutoff threshold cycle (*C*_*t*_) value of 40. When multiple targets were detected in a sample, the *C*_*t*_ values for those targets were averaged (**18**). When a single target was positive, the exact *C*_*t*_ value was used. A valid negative result for SARS-CoV-2 detection was determined by amplification of internal control using a cutoff *C*_*t*_ value of 30.

### 2.3. Statistical analysis

We considered a true positive sample to be any positive detected from either NPS or saliva. Starting with this definition, positive percent agreement (PPA) and negative percent agreement (NPA) were calculated using MedCalc Software Ltd.

Statistical analyses comparing different *C*_*t*_ values and days between the onset of symptoms and the test date were performed using a Mann–Whitney *U*-test.

## 3. Results

We randomly analyzed paired samples from 256 pediatric patients (*M* = 151 and *F* = 105, median age = 4.24 years) who were admitted to the hospital emergency room of AOUI, Verona, between September and December 2020. We analyzed this cohort by dividing patients into two subgroups as follows: Group A with patients between 0 and 6 years old (190 patients; 115 boys and 75 girls) and Group B with patients >6 years (66 patients; 36 boys and 30 girls). Paired NPS and saliva samples were collected from all patients and analyzed for the presence of the SARS-CoV-2 genome using the same extraction platform and amplification procedure.

SARS-CoV-2 genes were detected in 16 out of 256 overall NPS samples, whereas 13 out of 256 samples were positive in the SS samples. Demographic and clinical data were collected and are presented in Table 1. Unfortunately, we could not collect sanitary information about 6 out of 16 patients, but it is important to note that in this group of the positive sample, we presented two children of 1 month and 7 months old. Some patients were discharged after a short observation period (36 or 48 h, depending on age), and most of them were discharged soon after Emergency Department (ED) admission. Overall concordance between NPS and SS was detected in 253 out of 256 samples (98.83%). All data, classified into Groups A and B, are shown in Table 2.

**Table 1:** Anagraphical and sanitary information of positive patients.

**ID**	**Age**	**Reason for hospitalization**
1	1 y. 6 m.	Gastroenteritis
2	12 y. 0 m.	Asthma exacerbation
3	12 y. 2 m.	Suspected appendicitis
4	1 y. 4 m.	NA
5	1 y. 3 m.	Positive grandmother, skin rush
6	1 m.	Positive father, fever, and rinitis
7	1 m.	Positive father and rinitis
8	10 y. 8 m.	Positive father and abdominal pain
9	1 y. 7 m.	NA
10	8 y. 1 m.	NA
11	13 y. 5 m.	NA
12	3 y. 3 m.	Gastroenteritis
13	7 m.	Positive father and fever
14	5 y. 2 m.	NA
15	9 y. 7 m.	Incidental diagnosis
16	8 y. 3 m.	NA

NA, not applicable, for these patients, we have no information; y, years; m, month.

**Table 2:** Frequency of positive-paired NP and saliva swabs from all 256 patients enrolled in this study.

	**Mean age**	**Patients**			**All positive**	**NP positive swab**	**Salivary positive swab**	**Concordance between SS and NPS**
Group A (< 6 y. o.)	1.92	F	75	39.47%	5	5	5	
		M	115	60.53%	3	3	2	
*n*	190		8/190	8	7	87.50%
Group B (>6 y. o.)	10.92	F	30	45.45%	4	4	4	
		M	36	54.55%	4	4	2	
*n*	66		8/66	8	6	75.00%
Total	4.24	F	105	41.02%	9	9	9	
		M	151	58.98%	7	7	4	
*n*	256		16/256	16	13	81.25%

F, female; M, male; n, total patients of the subgroup; NPS, nasopharyngeal swab; SS, salivary swab.

SARS-CoV-2 genes were detected in 8 out of 190 NPS (4.21%), whereas 7 out of 190 SS (3.68%) were positive in Group A. A total of seven samples were positive both for NPS and SS. One sample was positive for NPS only, as indicated above. In Group B, 8 out of 66 NPS samples (12.12%) were positive, whereas 6 out of 66 SS (9.09%) were positive. All six SS-positive patients showed a paired positive detection of the SARS-CoV-2 genome in NPS.

Given that NPS is the gold standard to identify SARS-CoV-2 (Hong et al., 2020), we compared SS and NPS data (statistical results are displayed in Table 3). The sensitivity was 81.25% (95% CI: 54.35–95.95%), whereas the specificity was 100.00% (95% confidence interval (CI): 98.47–100.00%), with a 100.00% positive predictive value (PPV), 99.01% (95% CI: 97.30–99.64%) negative predictive value (NPV), 99.05% (95% CI: 96.95–99.85%) accuracy, and 0.19 (0.07–0.52) negative likelihood ratio (NLR). The statistical analysis of Group A, following the same procedure for the whole number of samples as previously mentioned, indicated a sensitivity of 88.89% (95% CI: 51.75–99.72%), whereas the specificity was 100.00% (95% CI: 97.99–100%), with a 100% PPV, 99.52% (95% CI: 97.00–99.92%) NPV, 99.53% (95% CI: 97.22–99.99%) accuracy, and 0.11 (0.02–0.71) NLR. The statistical analysis for Group B indicated a sensitivity of 80% (95% CI: 44.39–97.48%), whereas the specificity was 100% (95% CI: 93.84–100%), with a 100% PPV, 97.32% (95% CI: 91.30– 99.21%) NPV, 97.58% (95% CI: 90.56–99.79%) accuracy, and 0.20 (95% CI: 0.06–0.69) NLR.

**Table 3:** Performance characteristics of paired test in groups A and B.

	**Prevalence**	**Sensitivity**	**Specificity**	**PPV**	**NPV**	**NLR**	**Accuracy**
Group A	4.20%	88.89% (51.75% to 99.72%)	100% (97.99% to 100%)	100%	99.52% (97.00% to 99.92%)	0.11 (0.02 to 0.71)	99.53% (97.22% to 99.99%)
Group B	12.12%	80% (44.39% to 97.48%)	100% (93.84% to 100%)	100%	97.32% (91,30% to 99.21%)	0.20 (0.06 to 0.69)	97.58% (90.56 % to 99.79%)
Total	5.07%	81.25% (54.35% to 95.95%)	100% (98.47% to 100%)	100%	99.01% (97.30% to 99.64%)	0.19 (0.07 to 0.52)	99.05% (96.95% to 99.85%)

Statistical analysis of disease prevalence, sensitivity, specificity, positive and negative predictive value (PPV and NPV), negative likelihood ratio (NLR), and accuracy of test were represented with their 95% confidence intervals.

**Figure 1:**
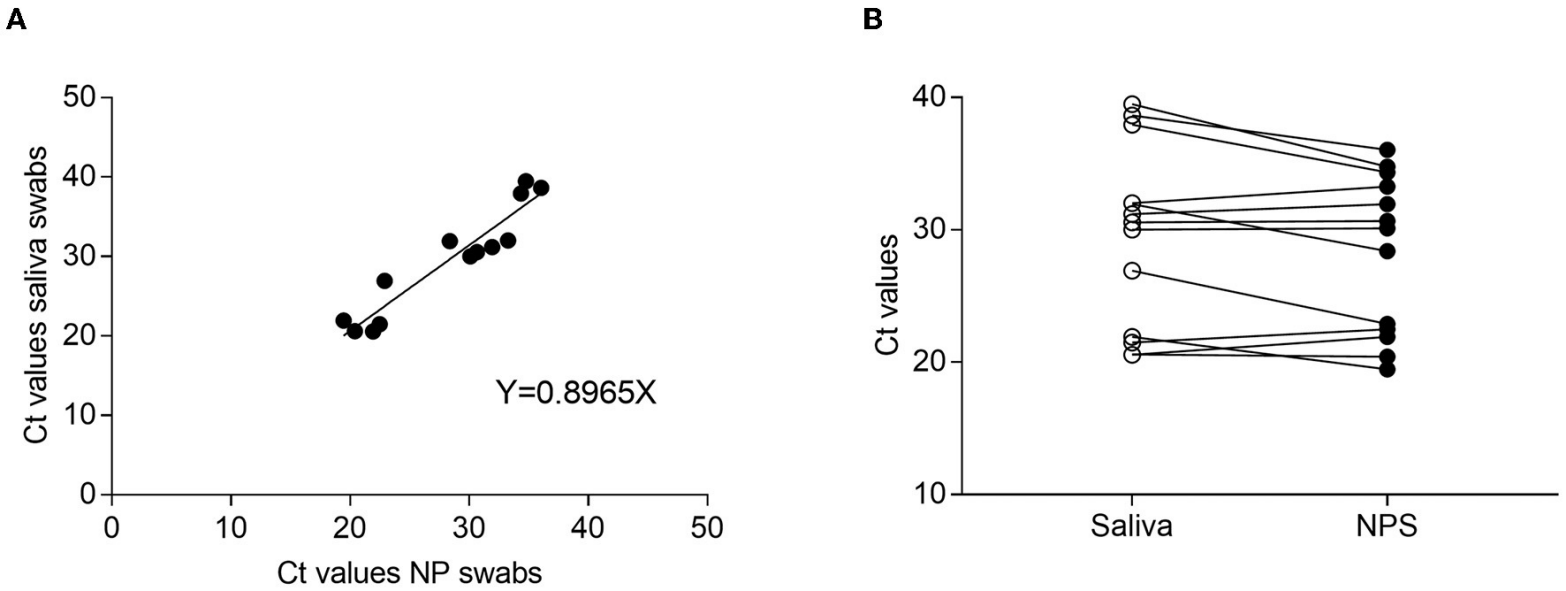
*C*_*t*_ comparison between NPS and saliva-paired samples. **(A)** Regression curve of *C*_*t*_ obtained from NPS and saliva samples. The graph shows a linear correlation between specimens with an *R*^2^ value of 0.8965. **(B)** Correlation between positive paired samples.

The differences between the *C*_*t*_ value average detected in NPS- and SS-positive samples were not statistically significant (*C*_*t*_ = 28.2 for NP vs. *C*_*t*_ = 29.48 for SS; *p* = 0.16, Wilcoxon matched pairs). The analysis of linear regression indicated a linear association between the mean *C*_*t*_ values obtained from NPS and SS, with *R*^2^ = 0.8965 (Figure 1A). In addition, analyses of the correlation between *C*_*t*_ values of positive paired specimens showed that the *C*_*t*_ values of salivary samples were slightly higher than NPS, although not significantly (Figure 1B).

**Figure 2:**
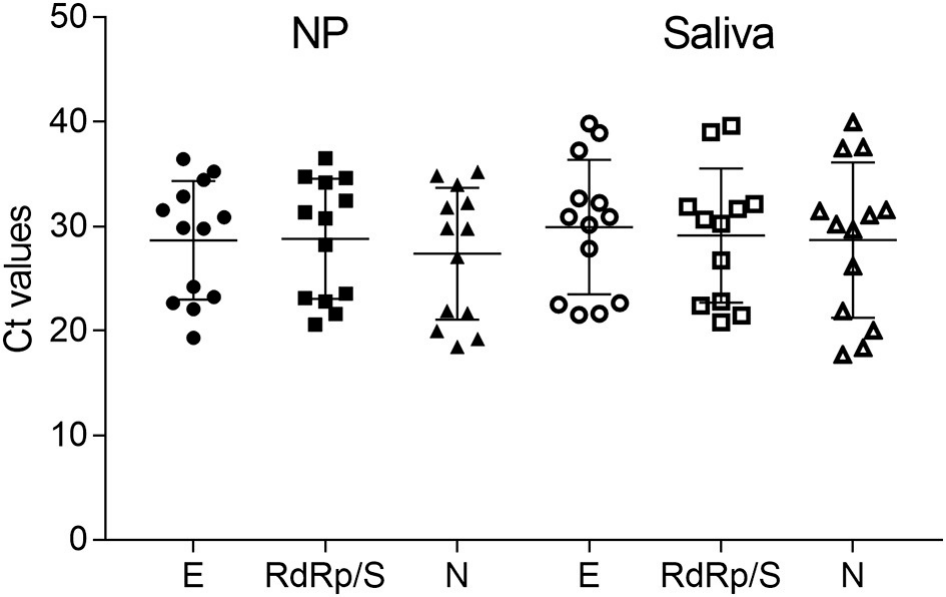
*C*_*t*_ values from paired samples distinct in *E*, RdRp/S, and *N* genes for both NP and saliva samples. The graph shows generally higher *C*_*t*_ values in saliva samples, but this difference is not significant.

To better evaluate the *C*_*t*_ value distribution, we compared the distinct *C*_*t*_ values of different targets obtained from paired samples (Figure 2). Although no significant differences were detected, *C*_*t*_ values of SS displayed a weak increase compared with NPS.

## 4. Discussion

In this study, we compared saliva sampling with classical NPS sampling in pediatric patients to determine the effectiveness of this procedure in the diagnosis of SARS-CoV-2 infection.

Our aim was to compare NPS and SS data in a cohort of pediatric patients, to better understand whether SS could be preferentially used in the pediatric population without losing specificity and sensitivity in viral genome detection. The use of less invasive methods to detect the SARS-CoV-2 virus, as an alternative to NPS collection, may be valuable, particularly for diagnosis in children, who represent a difficult category of patients for NPS collection. In addition, despite the small percentage of severe cases in children, they play an important role in the spread of viral infections in adults and during school attendance.

To achieve this goal, we selected NP and saliva-paired samples collected from pediatric patients admitted to the Pediatric Emergency Department in the last 4 months of 2020, during the second pandemic wave. Saliva collection was performed with a cotton swab, adopting a procedure where the operator rolls the swab in the mouth and under the tongue. Swab was preferred in children where the use of sponge and salivette could be dangerous (i.e., ingestion). To reduce differences in post-collection analysis, we used the same collection system (swab) and the same molecular protocol to detect the virus, namely a multiplex RT-PCR.

Analysis and comparison of these two different sample collections were performed in pre-school age children (< 6 years; Group A) and school-age children (>6 years; Group B).

Our findings suggest that the use of saliva sampling in children could be considered for pediatric patients instead of NPS. We observed an overall concordance between NPS and SS of 98.83% and a higher sensitivity in SS from children belonging to Group A (88.89%) with respect to Group B (80%), confirming the results of previous studies (Heald-Sargent et al., 2020; Al Suwaidi et al., 2021), which indicate the cause of higher viral load in this subpopulation. This study analyzes, for the first time, SS and NPS harvested from pediatric patients younger than 6 years old, differently from the previously published studies (Al Suwaidi et al., 2021). It is noteworthy that two of our positive samples belonged to one 1 month and one 7 months old patients. These data strengthen the results of the comparison between saliva and nasopharyngeal swabs, suggesting that saliva is a good alternative biological material for SARS-CoV-2 detection, especially for pediatric patients.

Multiplex RT-PCR performed on NP samples is reliable and sensitive, but the use of swabs for the collection of NPS samples is invasive and is not easy to perform, especially in pediatric patients. To overcome this drawback, some studies suggested the use of SS and several different tools to collect samples, including a sponge, salivette, or drooling into a tube. The use of saliva as a specimen for viral detection was already suggested for several RNA viruses including Ebola and Zika viruses (Niedrig et al., 2018; Gorchakov et al., 2019; Khurshid et al., 2019). This approach can even be applied to self-collection and does not require a medical operator to minimize contact with health operators.

When applied to SARS-CoV-2 infection, saliva samples showed somewhat discordant results, particularly between young and adult populations. Most previous studies demonstrated a good correlation between data obtained from NPS and SS, despite the different specificity and sensitivity (Williams et al., 2020; Al Suwaidi et al., 2021; Jamal et al., 2021). This discrepancy in SS data could be due to different collection and storage procedures, different collection times during the day, variation in viral concentration if patients coughed recently or ate or drank before collection, and finally, the stage of the disease at which the operator collected the SS. Indeed, Zhu et al. (2020) demonstrated that the viral load peak in SS during the first week of infection is followed by a time-dependent decrease in patients with mild and severe COVID-19. Furthermore, we must consider that, in some studies, the SS was collected by the patients themselves, so the sampling method may have been inadequate.

In conclusion, our findings suggest that saliva samples could be a valuable alternative to NP sampling in pediatric patients. The relatively non-invasive collection method, the ease of sample storage and transport, and a lower discomfort compared to NPS represent favorable features that could promote the wider use of saliva sampling in children.

## Data availability statement

The datasets presented in this article are not readily available because all relevant data are contained within the article. Requests to access the datasets should be directed to davide.gibellini@univr.it.

## Ethics statement

The studies involving human participants were reviewed and approved by University of Verona Ethics Committee protocol 3442 CESC (Comitato Etico per la Sperimentazione Clinica). Written informed consent from the participants' legal guardian/next of kin was not required to participate in this study in accordance with the national legislation and the institutional requirements.

## Author contributions

DS: conceptualization and review. ED, VL, and AL: methodology, formal analysis, writing, and editing—original draft. LB: methodology and review. NM and PB: methodology. DG: funding acquisition, conceptualization, writing, and review. All authors read and approved the final manuscript.
